# The Speed of Visual Discrimination Differs between Foveola and Perifovea: A Combined EEG and Behavioral Investigation

**DOI:** 10.1523/ENEURO.0078-25.2025

**Published:** 2025-08-08

**Authors:** Alessandro Benedetto, Samantha K. Jenks, Maruti V. Mishra, Barry Giesbrecht, Martina Poletti

**Affiliations:** ^1^Department of Brain and Cognitive Sciences, University of Rochester, Rochester, New York 14627; ^2^Center for Visual Science, University of Rochester, Rochester, New York 14627; ^3^Department of Neurosciences, Psychology, Drug Research and Child Health (NEUROFARBA), University of Florence, Florence 50121, Italy; ^4^Department of Psychology, California State University, Bakersfield, California 93311; ^5^Department of Psychological and Brain Sciences, University of California, Santa Barbara, California 93106

**Keywords:** eccentricity, EEG, N1, reaction time, speed, visual processing

## Abstract

Despite the vivid experience of homogeneous vision, our visual system is inherently endowed with highly inhomogeneous structures. Although the temporal characteristics of visual responses vary with eccentricity, the connection between this variation, the speed of visual processing, and its underlying neurophysiological mechanisms remains a topic of debate. Here, we performed simultaneous recordings of high-precision gaze positions and EEG activity to investigate how foveal and perifoveal stimulations impact reaction times (RTs) and visual evoked potentials (VEPs). Volunteers discriminated the position and orientation of a U-shaped figure with the aperture facing either upward or downward. Stimuli were presented briefly (50 ms) either in the foveola (0.33°) or perifovea (6.5°), to the right or left of the fixation point. Stimulus size in the perifovea condition was adjusted according to the cortical magnification factor (stimulus size: 0.2° and 0.75° for the foveola and perifovea conditions, respectively). When stimuli were equated for sensitivity and cortical area of stimulation, we observed faster RTs in the perifovea condition (16.8 ± 4 ms) compared to the foveola. The analysis of the VEP revealed a similar effect for the N1 response (11.0 ± 4 ms), a parieto-occipital component associated with discriminative processing and influenced by spatial attention. Overall, our findings suggest that visual discrimination speeds vary across eccentricities, with faster processing and shorter latency of early visual responses in the perifovea compared to the foveola.

## Significance Statement

This study investigates how visual processing speeds vary with eccentricity. By recording high-precision gaze positions and EEG activity, we asked volunteers to discriminate the orientation of a U-shaped figure briefly presented foveally or perifoveally. We found that visual stimuli presented perifoveally led to faster reaction times and shorter visual evoked potentials compared to a foveal presentation. In particular, reaction times for perifoveal stimuli were faster by 16.8 ± 4 ms, with similar effects observed in the N1 visual evoked potential component (11.0 ± 4 ms). These findings suggest that early visual processing is quicker in the perifovea, highlighting the presence of effective temporal inhomogeneities across the retina.

## Introduction

While it is well-established that many aspects of vision deteriorate with increasing eccentricity from the center of gaze, differences in the temporal dynamics of visual processing between the central and peripheral vision have been less studied. Interestingly, eccentricity-dependent structural and physiological differences in the architecture of the visual system (Schultze, 1866; Østerberg, 1935; Curcio et al., 1990) not only lead to spatial processing differences, such as reduced visual resolution (Anstis, 1974; Rovamo and Virsu, 1979), greater crowding (Bouma, 1973; Toet and Levi, 1992; Snowden and Hammett, 1998), and lower contrast sensitivity (Robson and Graham, 1981; Pointer and Hess, 1989) for peripheral versus central stimuli, but they could likely lead to differences in the temporal dynamics of visual processing.

The human visual system comprises various classes of neurons distinguished by both function and morphology. A broad primary distinction is observable in the retinal ganglion layer, where large parasol ganglion cells project to the magnocellular layers of the lateral geniculate bodies and smaller midget ganglion cells project to the parvocellular layers (Leventhal et al., 1981). This difference is evident not only in the spatial frequency (SF) selectivity, with magnocells and parvocells preferring low and high SFs (Croner and Kaplan, 1995), respectively, but also in temporal responsiveness. Magnocells are characterized by a conduction speed and integration time approximately 20 ms faster than parvocells (Nowak and Bullier, 1997; Schmolesky et al., 1998; Lamme and Roelfsema, 2000). These temporal differences have significant implications for behavior. Numerous psychophysical studies have indeed shown that simple manual reaction times (RTs) for low SFs, driven primarily by magnocells, are faster than RTs for high SFs, primarily driven by parvocells (Breitmeyer, 1975; Smith, 1995; Murray and Plainis, 2003).

In particular, the parvo-to-magno cell ratio decreases with retinal eccentricity (Connolly and Van Essen, 1984; Schein and de Monasterio, 1987; Azzopardi et al., 1999) (but see also Livingstone and Hubel, 1988). This phenomenon likely introduces heterogeneity in the temporal dynamics of the neural responses across the visual field. Furthermore, even within the same neuron type, such as midget ganglion cells and cones, the neuronal temporal response function undergoes substantial changes based on eccentricity, leading to responses approximately twice as slow in the fovea compared to the periphery (Sinha et al., 2017).

Consistent with this neurophysiological evidence, numerous studies demonstrated a higher temporal sensitivity in the periphery compared to the fovea (Hartmann et al., 1979; Tyler, 1987; Solomon et al., 2002) (but see also Rovamo and Raninen, 1984). Surprisingly, however, the evidence from the RT studies is inconsistent. For instance, after addressing the RT differences related to changes in visual discriminability, decision criteria, and correction for cortical magnification, Carrasco et al. (2003) demonstrated that the processing speed of oriented Gabor patches was approximately 50 ms faster for stimuli presented at 9° than 4° eccentricity. A similar effect was also observed for foveola (0°) versus parafovea (3°) stimulation (Poletti et al., 2017), suggesting a generalized effect across all eccentricities. Remarkably, this relationship is evident not only for manual but also for oculomotor RTs; saccadic eye movements exhibit longer latencies for stimuli presented in the foveola (<1°) compared to further eccentricities (Wyman and Steinman, 1973; Kowler and Anton, 1987; Kalesnykas and Hallett, 1994; Poletti et al., 2020). Yet, even if some studies have shown faster processing times for stimuli presented in the periphery compared to the fovea (Carrasco et al., 2003, 2006; Poletti et al., 2017), others have reported increased RTs with increasing retinal eccentricities (Rains, 1963; Osaka and Yamamoto, 1978; Carrasco et al., 1995; Wolfe et al., 1998; Staugaard et al., 2016).

A more direct approach to estimating the effect of eccentricity on the latency of neuronal impulse responses is to compare the EEG activity evoked by stimuli presented at different eccentricities. Indeed, neural latency variation is a major contributor to variation in motor latency (Lee et al., 2016), and numerous studies have shown that RTs can be predicted by the latencies of EEG visual evoked components. In particular, the latency of the N1 visual component, which is thought to reflect the operation of discrimination processes (Vogel and Luck, 2000) and to be modulated by spatial and object-based attention (Martinez et al., 2007), has been demonstrated to predict single-trial and within-subject variability in RTs (Wastell and Kleinman, 1980; Antonova et al., 2016; Ribeiro et al., 2016).

Interestingly, the relationship between the N1 latency and the eccentricity is less clear. Some studies have reported shorter N1 latency for stimuli presented in the periphery than in the fovea (Baseler and Sutter, 1997; Kremláček et al., 2004; Hagler, 2014), while others reported mixed or null effects (Busch et al., 2004; Handy and Khoe, 2005; Laron et al., 2009; Capilla et al., 2016). This variability among studies may stem from technical caveats known to modulate the latencies of evoked potentials and reaction times, as well as differences in the experimental paradigm. Factors such as differences in task difficulty between eccentricities or unmatched cortical areas of stimulation could contribute to these discrepancies. Also eye movements, including fixational eye movements (e.g., microsaccades and ocular drift), if not accounted for, can significantly influence both perceptual performance (Poletti et al., 2013; Shelchkova and Poletti, 2020) and EEG responses (Yuval-Greenberg et al., 2008; Dimigen et al., 2009), further complicating the interpretation of EEG findings in studies investigating eccentricity-related effects.

Here we examined differences in the temporal dynamics of visual discrimination between foveolar and extrafoveal vision. To elucidate the relationship between the timing of visual discrimination and the stimulus eccentricity, we rigorously controlled for task difficulty, cortical area of stimulation, and fixational eye movements, and we measured choice RTs and N1 latencies for stimuli presented either in the foveola (0.33°) or the perifovea (6.5°). Our results revealed that both RTs and N1 latencies were faster for perifoveal compared to foveolar stimulation. Overall, our findings suggest that visual discrimination speeds exhibit variation across eccentricities, with faster processing and shorter latency of early visual responses observed for more peripherally presented stimuli.

## Materials and Methods

### Participants

**Figure 1.**
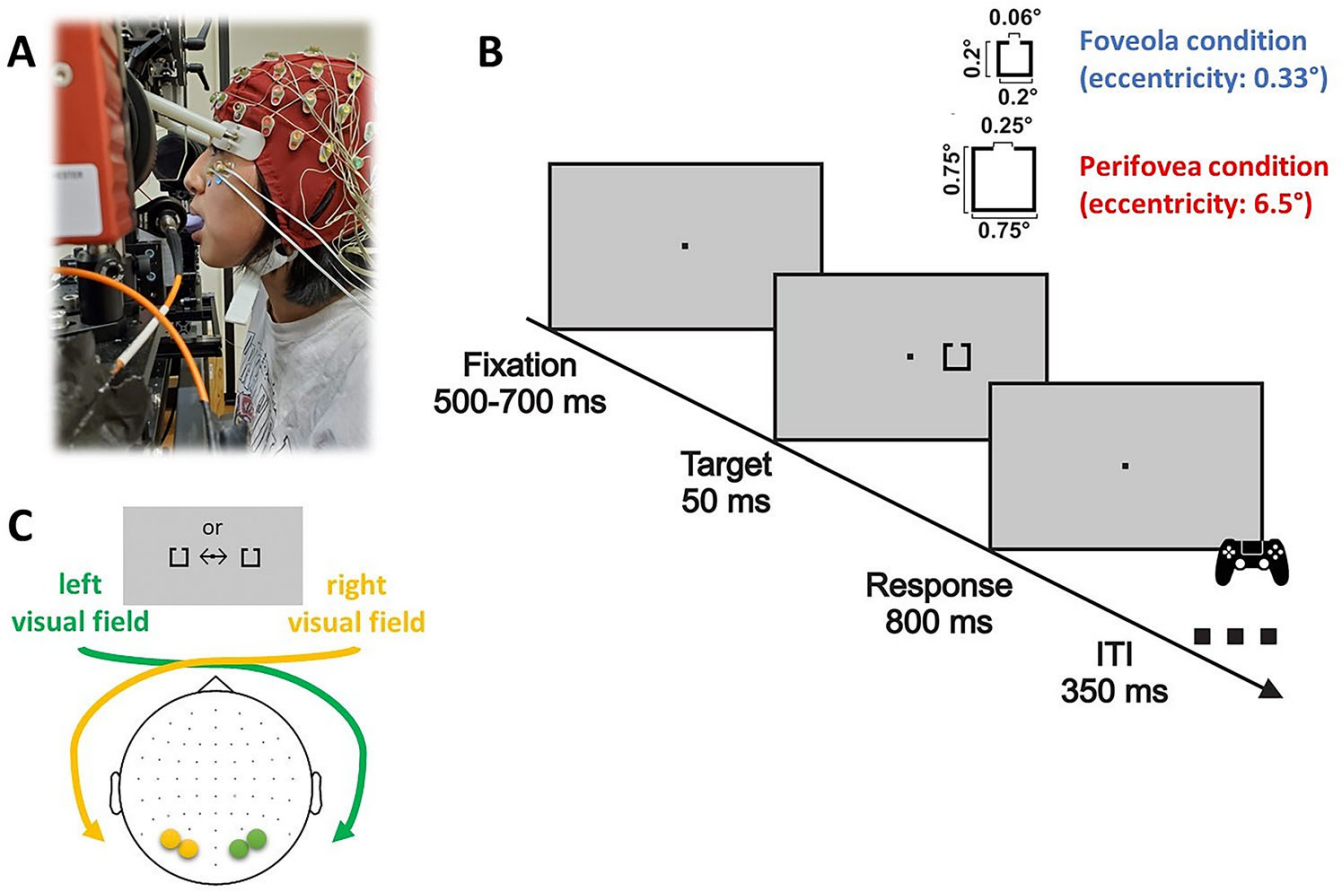
Experimental setup and procedure. ***A***, Example of the experimental setup. Eye movements and EEG were acquired simultaneously. To assure a stable position, the head was stabilized by means of a bite-bar and plastic head holder. ***B***, After a random delay (500–700 ms), the stimulus briefly appeared on the left or right visual field and, block-wise, at different eccentricities: either in the foveola (0.33°) or in the perifovea (6.5°). Participants reported the orientation of the stimulus’ aperture (up/down) and the position (left/right) of the stimulus by pressing the corresponding button on a joy-pad. ***C***, VEPs were estimated on the parieto-occipital electrodes (PO3–PO7 for the right visual field or PO4–PO8 for the left visual field stimulation).

A total of 19 subjects, including two of the authors (24 ± 4 years old, mean age and ±1 standard deviation (SD), 6 males and 13 females), participated in the study. One participant took part only in the behavioral part of the experiment, while three participants were excluded from the EEG analysis due to inadequate signal quality, preventing a reliable assessment of the evoked response, particularly the N1 latency (for details, see the following section in “*Data analysis*: *N1 peak latency: main analyses*”). For the additional single-trial analysis (see “*Data analysis*”), two participants were further excluded because it was not possible to reliably estimate the single-trial N1 component latency due to an excessively prominent alpha activity. Nineteen participants completed the behavioral experiment (Fig. 1), 15 completed the main EEG experiment, and EEG data for 13 of those subjects were used for the single-trial analysis. Additionally, seven volunteers from the behavioral experiment also took part in a control behavioral experiment. Notably, since the purpose of this control experiment was to test the robustness of the effect to task manipulations, the volunteers were selected among those who showed—in the main experiment—significant faster RTs in the perifovea than in the foveola. The experimental procedures are in line with the Declaration of Helsinki and followed the ethical procedures approved by the Research Subjects Review Board at the University of Rochester. Informed consent was obtained from all participants.

### Apparatus

Stimuli were displayed on a gamma-calibrated LCD monitor (ROG SWIFT 360 Hz PG259QN; monitor resolution: 1980 × 1080 pixels; refresh rate: 360 Hz), placed 83 cm in front of the observer in a dimly illuminated room. Stimuli were viewed binocularly. A dental imprint bite-bar and head-rest minimized head movements and maintained the observer at a fixed distance from the display. Vertical and horizontal eye position data were measured by means of a digital Dual Purkinje Image eye-tracker (sampling rate: 1 kHz; Fig. 1*A*) (Wu et al., 2023). Stimuli were rendered by means of EyeRIS, a hardware/software system for gaze-contingent display control that enables precise synchronization between the eye movement data and the refresh of the image on the monitor (Santini et al., 2007). Electrophysiological activity was continuously recorded at 2,048 Hz using a 64 channel ActiveTwo Biosemi system. The EEG offsets were checked before each block and kept below 20 μV. The synchronization between the EEG activity, the eye position, and visual stimulation was assured by means of two photocells triggering the onset of the stimulation period to both the Biosemi and the EyeRIS systems.

### Stimuli and procedures

Stimuli consisted of black squares outlines, with a small aperture oriented upward or downward. Stimulus size and aperture were scaled according to a cortical magnification factor (Rovamo and Virsu, 1979; Virsu and Rovamo, 1979), resulting in square with sides of 0.2° and 0.75°, and apertures of 0.06° and 0.25° for the foveola and perifovea conditions, respectively (Fig. 1*B*).

Each trial began with the appearance of red fixation dot (0.15°) at the center of the display on a gray background . After a random delay from 500 to 700 ms, the stimulus was flashed for 50 ms in the foveola (0.33°) or perifovea (6.5°), either to the right or left of the fixation. Stimulus eccentricity was kept constant within each block and participants were informed in advance about the eccentricity tested in each block. The order of blocks was pseudo-randomized, and the side of stimulus presentation (left or right) varied randomly within each block. Participants were instructed to maintain fixation and report, as soon as possible, the orientation (up/down) and the position (left/right) of the square by pressing the corresponding button on a remote controller (four-alternative forced choice (4AFC)). Motor responses on the controller buttons were spatially matched to the position and aperture of the stimulus, so that the hand to use for providing the response matched with the position of the stimulus on the screen.

We ran three control experiments: (1) an exact re-test of the main experiment (re-test blocked 4AFC); (2) an interleaved version of the main experiment, in which the two eccentricities were randomly interleaved within each block (interleaved 4AFC); (3) same as (2) but with an easier motor implementation, in which subjects were required to report the orientation of the stimulus aperture, irrespective of its left/right position, with the right hand (interleaved 2AFC).

Before each block, participants underwent a two-stage calibration procedure. In the first phase, they sequentially looked at each of the nine points of a standard 3 × 3 grid. Parameters were then refined in a second gaze-contingent phase, in which participants manually fine-tuned the estimated position of gaze, displayed in real-time on the monitor for each of the grid points. To counteract possible misalignment caused by drifts in the apparatus and/or minute head movements, the gaze-contingent procedure was repeated for the fixation point every trial.

### Data analysis

#### Eye movement preprocessing

All trials were screened for eye movements. Only trials with no (micro-)saccades and with and average gaze position within ±10’ from the central fixation marker were included in the main analyses. For the behavioral analyses, the average gaze position was estimated between ±50 ms around stimulus presentation. As eye movements generate long-lasting response that might affect the EEG activity, the gaze estimation window for the visual evoked potential (VEP) analyses was raised to ±200 ms from stimulus presentation. This resulted in 77 ± 16 and 59 ± 18% (mean ±1 SD) of trials without eye movements, for the behavioral and EEG analysis, respectively.

#### Reaction times and sensitivity measures

We estimated—for each participant—the median RT for both the foveola and perifovea condition. RTs were only estimated on trials with correct responses, and with no eye movements. This resulted—for each tested eccentricity and participant—in 500 ± 223 trials (mean ±1 SD), for the main experiment. Control experiments resulted in 65 ± 12 (blocked 4AFC re-test), 68 ± 13 (interleaved 4AFC), and 57 ± 18 trials (interleaved 2AFC). In addition to standard group statistics (i.e., ANOVA), we also compared—within each participant—the median RTs at the two tested eccentricities by means of a two-sided Wilcoxon rank sum test. Visual sensitivity (d-prime or d’) was estimated from the proportion of correct responses and adjusted to the number of alternative forced choices (Hacker and Ratcliff, 1979). To test—within each participant—the sensitivity differences at the two tested eccentricities, we ran a bootstrap sign-test (1,000 simulations, with replacement) to compare the two resulting d-prime distributions. The *p*-values of the distribution differences were obtained from the proportion of simulations in which the d’ in the perifovea was higher than in the foveola (two-sided, *α* = 0.025). In addition to the standard frequentist analyses, we report Bayes factors (BF_10_) for *t*-tests and correlations. For *t*-tests, a Cauchy prior with a scale factor of 0.707 was used. Bayes factors provide a measure of the strength of evidence in favor of the alternative hypothesis (H_1_) compared to the null hypothesis (H_0_). For example, BF_10_ values below 0.333 are conventionally interpreted as evidence in favor of the null hypothesis (H_0_), while values above 3 indicate increasing support for the alternative hypothesis (H_1_), facilitating a more nuanced interpretation of the results.

#### EEG

##### EEG preprocessing

EEG analyses were performed with the MATLAB toolbox EEGLAB (Brunner et al., 2013) and FieldTrip (Oostenveld et al., 2011), in combination with the plugins *firfilt, cleanLine*, and custom code. The EEG data were first referenced to the central midline electrode and down-sampled to 512 Hz. The reference and the noisy channels were then automatically removed (rejection criterion: normalized kurtosis >5). The signal was high-pass filtered (cutoff of 0.2 Hz, Blackman sinc FIR filter with a transition bandwidth of 0.4 Hz), line noise was removed (function *cleanline*), and recordings were concatenated. Rejected channels were interpolated (spherical interpolation), and the EEG was re-referenced to a common average (excluding EOG channels). Finally, EEG data were epoched around the time of stimulus onset. Baseline activity was defined as the average activity in the 150 ms before stimulus onset. A low-pass filter (35 Hz) was applied to the VEPs. Main analyses, after discarding incorrect trials and trials with eye movements, were based—for each participant and eccentricity—on 382 ± 167 trials (mean ±1 SD).

##### N1 peak latency: main analyses

The VEP was first independently computed for stimuli presented in the left or right visual field, utilizing contralateral electrodes predetermined a priori (PO3–PO7 for the right visual field or PO4–PO8 for the left visual field stimulation; Fig. 1*C*), followed by averaging. The N1 latency was determined as the local negative peak occurring between 100 and 300 ms, with a peak prominence exceeding 1 μV. Following this automated procedure, a visual inspection was performed for confirmation. In three participants, the N1 peak prominence was below the a priori defined threshold of 1 μV in foveal or peripheral stimulation conditions, preventing a reliable assessment of the evoked response. Therefore, they were excluded from the analysis (resulting in 15 participants). See also Extended Data Figures 3-1:6 for additional analyses on the N1 peak latencies and individual curves, including ipsilateral N1 latencies, lateralized N1 latencies, and global mean field power latencies.

##### N1 latency: single-trial analyses

We implemented a template-matching procedure to measure the single-trial N1 latency, employing a step-wise approach. Initially, for each epoch, the channel’s data were normalized (z-scored). A low-pass filter was then applied at 15 Hz. Baseline within the time window of −150 to 0 ms was removed, and a kernel signal was estimated for each participant. The kernel was obtained from averaging the VEPs from the two parieto-occipital electrodes contralateral to the stimulation site (PO3 and PO7, or PO4 and PO8 for the right and left visual field stimulations, respectively), from 50 to 250 ms. Subsequently, cross-correlation between the single-trial VEP (as the average of the two electrodes contralateral to the stimulation side) and the kernel was performed. Only trials in which the maximal coefficient of correlation value (i.e., the point of maximal similarity between the VEP and the kernel) surpassed 0.7, and in which its lag fell within the range of –35 to 35 ms (i.e., allowing a maximal temporal shift of 70 ms) , were kept for further analyses. N1 latency was then estimated for each trial, as in described earlier. For the correlation analysis, outliers in either RT or latency were removed, as well as the main effect of stimulation eccentricity (i.e., partial correlation: *ρ*_*xy*·*z*_). Outliers were identified as elements surpassing 1.5 interquartile ranges above the upper quartile (75%) or below the lower quartile (25%). Overall, the procedure resulted—for each participant—in 154 ± 70 and 117 ± 68 trials (mean ±1 SD) for the foveola and perifovea conditions, respectively.

## Results

**Figure 2.**
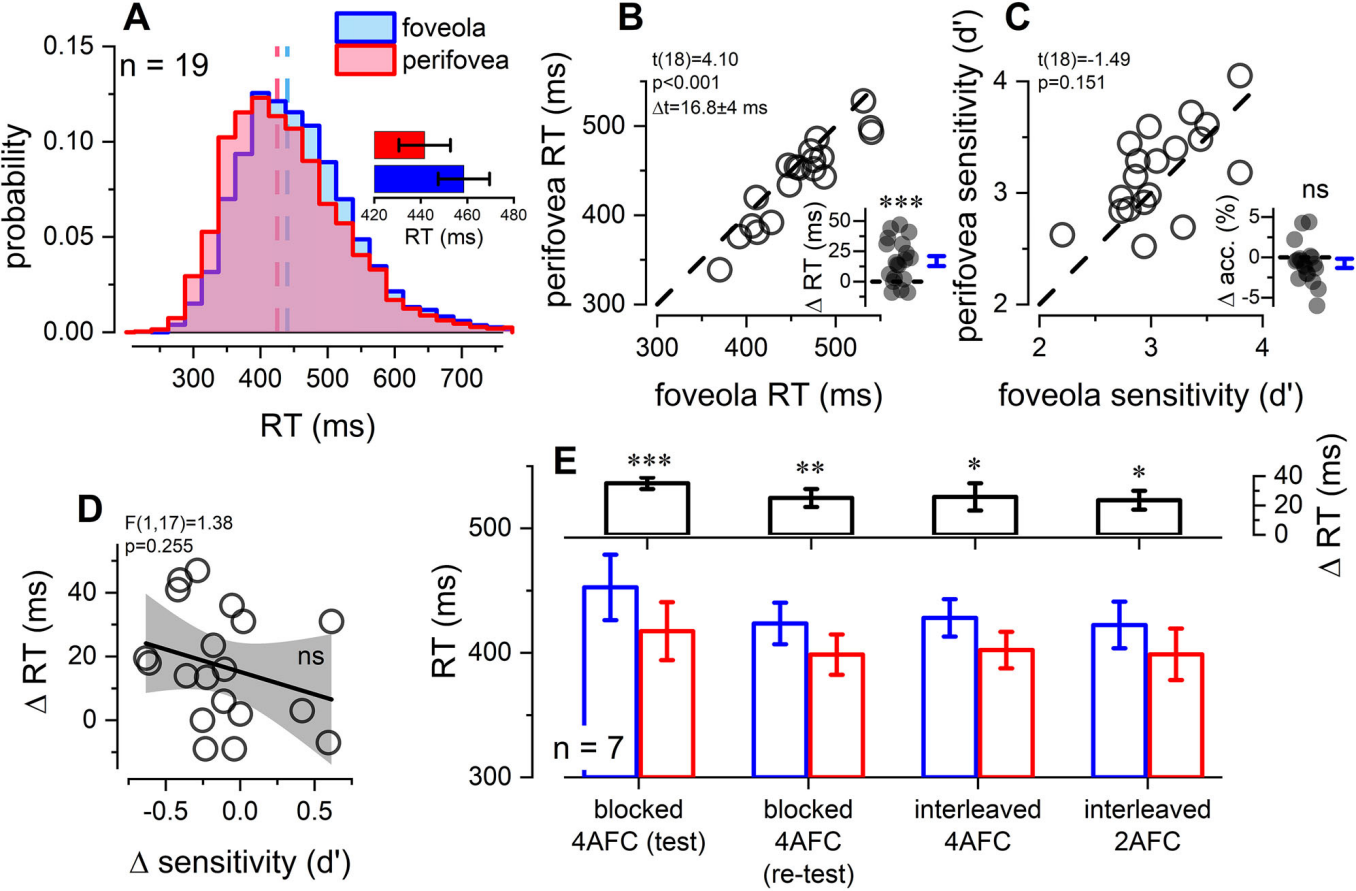
Behavioral results: RTs. ***A***, Aggregate observer RT probability distribution for the foveola (blue) and perifovea (red) stimulation. RT is about 17 ms faster in the foveola than in the perifovea. The bar plot shows the RT (mean ±1 sem) for the two tested eccentricities. ***B***, Scatter plot showing individual median RT for the foveola (*x*-axis) and perifovea (*y*-axis) presentation. Each dot represents a single participant. Most of the dots scatter below the equality line (dashed line) indicating that RTs are faster in the perifovea than in the foveola (*p* < 0.001). The inset shows the difference distribution in RT. Blue bar reports the mean RT difference (±1 SEM). ***C***, Same as in (***B***) but for sensitivity (d-prime). Sensitivity did not vary between the two eccentricities (*p* = 0.151). ***D***, Linear regression analysis reveals that the difference in RT (*y*-axis) does not correlate with the difference in sensitivity (*x*-axis, *p* = 0.255). Thick line and gray area show the best linear regression and 95% confidence intervals, respectively. ***E***, Bar plot reporting the average (±1 SEM) RT for a subset of volunteers (*n* = 7). The bar plot shows the results from the main experiment (blocked 4AFC—test), its re-test several weeks after (blocked 4AFC—re-test), and for two interleaved versions of the same experiment (interleaved 4AFC and interleaved 2AFC). Choice RTs for stimuli presented in the foveola (blue) were longer than in the perifovea (red), irrespective of the task design (blocked vs interleaved) and discrimination/motor difficulty (2AFC vs 4AFC). The bar plot on the top shows that the RT eccentricity difference is significant for all tasks. Asterisks mark statistical significance: 0.05 > * > 0.01 > ** > 0.001 > ***, *p*-values are Bonferroni–Holm corrected.

We first investigated the behavioral effect of eccentricity on RTs. In 19 volunteers, we measured choice RTs to stimuli presented in the foveola and perifovea. Figure 2*A* shows the RTs distribution at the two eccentricities. We compared, for each participant, the median RT at the two eccentricities. The scatter plot in Figure 2*B* shows that most participants exhibited a faster RT, of 16.8 ± 4 ms (mean ± 1 SEM) on average, when stimuli were presented in the perifovea compared to the foveola (two-tailed paired *t*-test: *t*(18) = 4.10, *p* < 0.001, BF_10_ = 52.242). Notably, 15 out of 19 participants had faster RTs in the perifovea than in the foveola, with this effect being significant at the single-subject level in 10 of those subjects (two-sided Wilcoxon rank sum test for equal medians, *p* < 0.05).

To ensure that this reaction time effect was not driven by differences in task difficulty, we compared response accuracy in the two conditions. Subjects’ accuracy was overall very high (around 95 and 96% of correct responses for the foveola and perifovea stimulations, respectively) and visual sensitivity (d-prime) did not vary between the two eccentricities (*t*(18) = 1.49, *p* = 0.151, BF_10_ = 0.611; Fig. 2*C*), indicating that overall the difficulty between the two conditions was balanced. To verify that the effect of RT was not related to the inter-individual differences in accuracy, we analyzed the correlation between RTs and sensitivity. Results showed that the differences in RT and d’ between the two conditions were not correlated (*F*(1, 17) = 1.38, Pearson’s *ρ*(19) = −0.274, R^2^ = 0.075, *p* = 0.255, BF_10_ = 0.331; Fig. 2*D*), nor were individual RTs and d’ (*F*(1, 36) = 2.09, Pearson’s *ρ*(19) = −0.234, R^2^ = 0.055, *p* = 0.156, BF_10_ = 0.277; not shown). This suggests that task difficulty was well balanced between the two eccentricities and the differences in RTs were not arising from differences in visual sensitivity.

We re-tested after several weeks from the main experiment seven volunteers who originally showed a significant eccentricity effect in the main experiment, using the same procedure as the main experiment (i.e., block design, 4AFC). The correlation between RTs of the test and re-test was strong (*F*(1, 12) = 119, Pearson’s *ρ*(7) = 0.953, R^2^ = 0.91, *p* < 0.001, BF_10_ = 57.185). In this subset of participants, we additionally tested different combinations of variants of the original procedure (blocked vs interleaved design, with 2AFC vs 4AFC). Overall, this results in four task conditions: (1) original blocked 4AFC, (2) re-test blocked 4AFC, (3) interleaved 4AFC, and (4) interleaved 2AFC (Fig. 2*E*). The 2 × 4 repeated measure ANOVA revealed the main effect of eccentricity (*F*(1, 6) = 31.356, *p* = 0.001). Neither the main effect of task nor the eccentricity-task interaction was significant (task: *F*(3, 18) = 2.27, *p* = 0.114; interaction: *F*(3, 18) = 0.97, *p* = 0.427). Post-hoc comparisons revealed that—for all tasks—RTs were significantly faster in the perifovea as compared to the foveola (*p* < 0.05, Bonferroni–Holm corrected; see the top panel in Fig. 2*E*), with a comparable effect size across the different task conditions (Δ*t* ± 1 SEM for all four task conditions: 35 ± 4, 25 ± 6, 25 ± 9, 23 ± 6 ms). Overall, the RT difference was stable over time and was not influenced by task procedure, indicating that the effect was robust.

Overall, the behavioral results demonstrate that choice RTs to stimuli presented in the perifovea (6.5°) were approximately 17 ms faster than in the foveola (0.33°). This effect, although relatively modest in size (corresponding to a shift of about 5%), is very robust: it is stable over time and is not modulated neither by the spread of spatial attention (as manipulated in the block vs interleaved design) nor by the discrimination/motor difficulties of the task (as manipulated in the 2AFC vs 4AFC).

**Figure 3.**
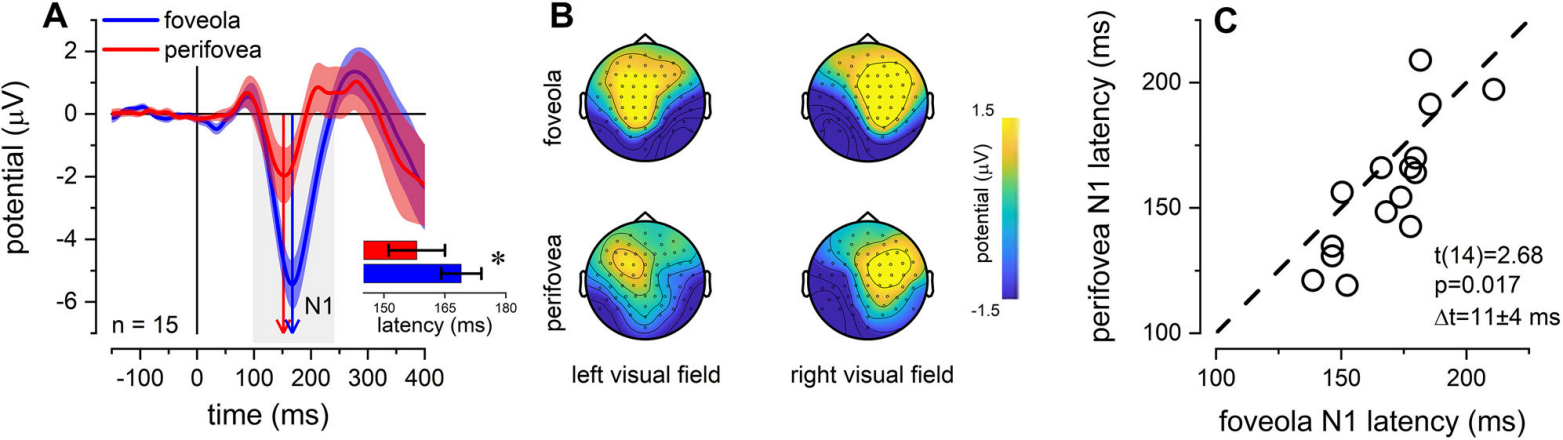
VEP results: N1 latency. ***A***, Grand-average VEP in the foveola (blue) and perifovea (red). Shadow areas mark 1 SEM. Vertical arrows show the N1 peak latency for the two eccentricity conditions. The inset reports the average N1 latency (±1 SEM) for the foveola (blue) and perifovea (red) stimulation. Asterisks mark statistical significance: 0.05 > * > 0.01. ***B***, VEP amplitude topographic distribution in the interval ±25 ms around the average N1 peak latency (140 to 190 ms from stimulus onset). The topoplots are shown for stimuli presented in the foveola (top panels) and perifovea (bottom panels), presented in the left visual field (left panels) and right visual fields (right panels). ***C***, Scatterplot showing the N1 peak latencies in the foveola (*x*-axis) and perifovea (*y*-axis). Each dot represents a single subject. The dots scatter below the unity line (dashed line), indicating that N1 latencies in the perifovea were shorter than in the foveola. Individual curves and additional analyses are reported in the Extended Data Figures 3-1:6.

We then estimated the effect of eccentricity on the latency of neuronal impulse responses, by comparing the EEG activity evoked by visual stimuli presented at different eccentricities. Figure 3*A* shows the grand-average VEP elicited by stimuli presented in the foveola (blue) and perifovea (red). The VEP estimated over the contralateral parieto-occipital electrodes revealed an early positive component peaking at about 90–100 ms (P1), followed by a prominent negative component (N1) peaking at about 150–160 ms from the stimulus onset. Here, we focused on the latency of the N1 visual component, thought to reflect the operation of discrimination processes (Vogel and Luck, 2000), and being associated with RTs (Wastell and Kleinman, 1980; Jaśkowski et al., 1990; Ribeiro et al., 2016). The topographic representation of the EEG activity in the ±25 ms around the average N1 peak latency (140 to 190 ms; Fig. 3*B*) revealed a clear dipole with a negative activity maximally expressed over the contralateral parieto-occipital electrodes, for both foveola and perifovea presentations. Similar to the results reported for RTs, the majority of participants exhibited a shorter N1 peak latency when the stimulus was presented in the perifovea compared to the foveola (*t*(14) = 2.68, *p* = 0.017, BF_10_ = 3.500; Fig. 3*C*), an effect of approximately 11 ± 4 ms. Notably, the RT analysis performed on the trials and participants included in the EEG response analysis showed an effect almost identical to that reported previously, and in the same ballpark, i.e., 17 ± 5 ms (*t*(14) = 3.33, *p* = 0.004, BF_10_ = 10.008)—as that observed for the N1.

10.1523/ENEURO.0078-25.2025.f3-1 Figure 3-1 **Contralateral VEP response.** Contralateral VEP responses in the foveola (blue) and perifovea (orange). Vertical bars indicate the N1 peak latency for the two eccentricity conditions (solid blue for the foveola and dashed orange for the perifovea). Each panel represents data from an individual subject. The N1 latency, for each subject and condition, was automatically determined using the MATLAB function findpeaks as the local negative peak occurring between 100 and 300  ms, with a peak prominence exceeding 1  V. In three participants (represented by panels with dark curves), the N1 peak prominence fell below the predefined threshold in either the foveal or peripheral stimulation conditions, preventing a reliable assessment of the evoked response. Consequently, these subjects were excluded from further analyses. Download Figure 3-1, TIF file.

10.1523/ENEURO.0078-25.2025.f3-2 Figure 3-2 **Ipsilateral VEP response.** Ipsilateral VEP responses in the foveola (blue) and perifovea (orange). Vertical bars indicate the ipsilateral N1 peak latency for the two eccentricity conditions (solid blue for the foveola and dashed orange for the perifovea). Each panel represents data from an individual subject. The ipsilateral N1 latency, for each subject and condition, was automatically determined using the MATLAB function findpeaks as the local negative peak occurring between 100 and 300  ms, with a peak prominence exceeding 1  V. In three participants (represented by panels with dark curves), the N1 peak prominence fell below the predefined threshold in either the foveal or peripheral stimulation conditions, preventing a reliable assessment of the evoked response. Consequently, these subjects were excluded from further analyses. Download Figure 3-2, TIF file.

10.1523/ENEURO.0078-25.2025.f3-3 Figure 3-3 **VEP results: ipsilateral N1 latency.** Scatterplot showing the ipsilateral N1 peak latencies in the foveola (x-axis) and perifovea (y-axis). Each dot represents a single subject. The dots scatter below the unity line (dashed line), indicating that ipsilateral N1 latencies in the perifovea were about 10  ms shorter than in the foveola (p = 0.028). Download Figure 3-3, TIF file.

**Figure 4.**
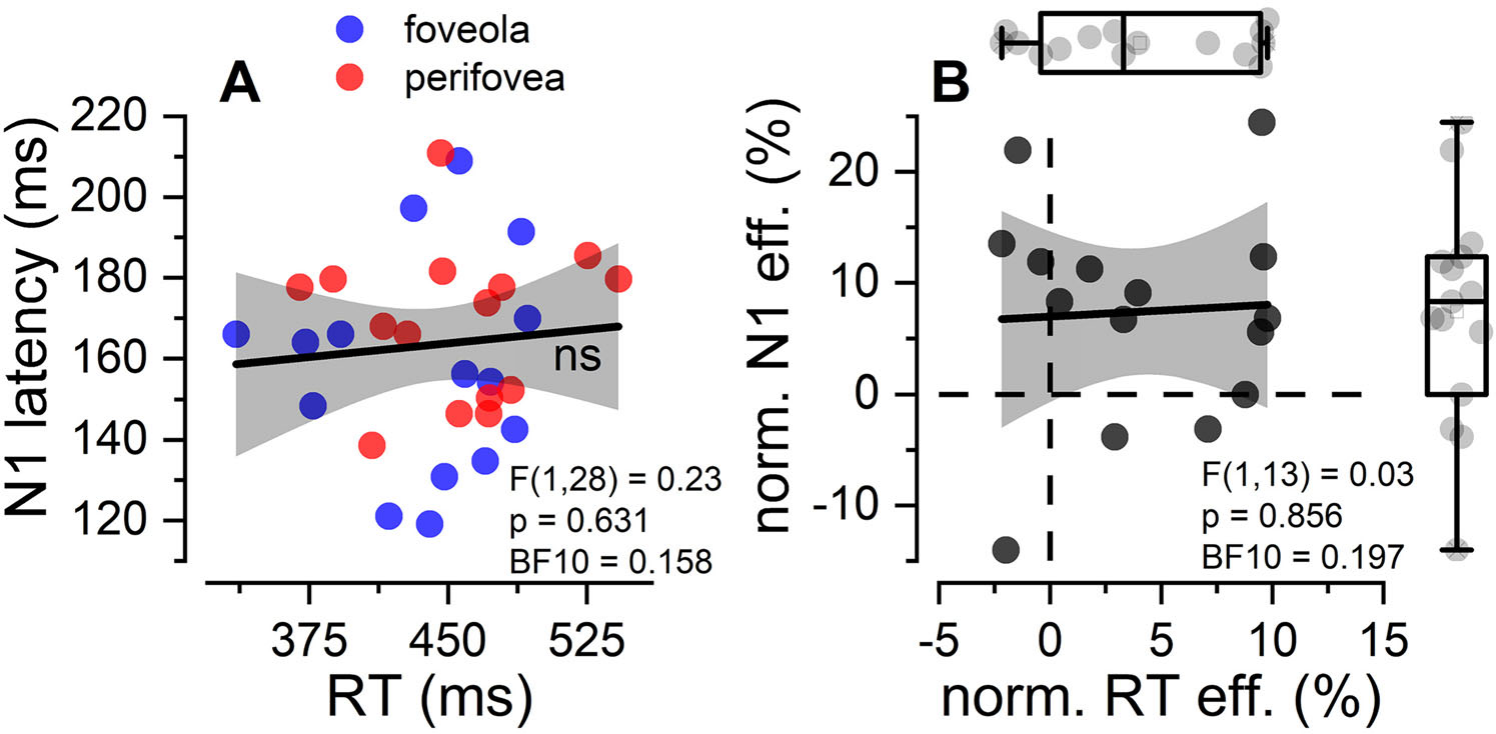
N1∼RT: correlational analysis. ***A***, Scatter plot showing the distribution of RT (*x*-axis) and N1 latency (*y*-axis), for both foveola (blue) and perifovea (red) conditions. Each dot represents the values obtained from a single participant for a single eccentricity condition. The linear regression between the two measures (black line) was not significant (*p* > 0.05). The shaded gray area reports the confidence interval of the linear regression analysis. ***B***, Scatter plot showing the normalized RT effect (*x*-axis) and N1 latency effect (*y*-axis). Similar to (***A***), there was no association between the two effects (*p* > 0.05). The shaded gray area reports the confidence interval of the linear regression analysis. Box plots display the distribution of the effects. The normalization of the effect was estimated as: $\frac{y=\frac{x_{foveola}\;-\;x_{perifovea}}{x_{foveola}\;+\;x_{perifovea}}}{2100}$; where *x*_*foveola*_ and *x*_*perifovea*_ are the RT and N1 latencies for the foveola and perifovea conditions, respectively.

10.1523/ENEURO.0078-25.2025.f3-4 Figure 3-4 **Lateralized VEP response.** Lateralized VEP responses in the foveola (blue) and perifovea (orange). Vertical bars indicate the lateralized N1 peak latency for the two eccentricity conditions (solid blue for the foveola and dashed orange for the perifovea). Each panel represents data from an individual subject. The lateralized N1 latency, for each subject and condition, was automatically determined using the MATLAB function findpeaks as the local negative peak occurring between 100 and 300  ms, with a peak prominence exceeding 1  V. In one participant (represented by panels with dark curves), the N1 peak prominence fell below the predefined threshold in either the foveal or peripheral stimulation conditions, preventing a reliable assessment of the evoked response. Consequently, this subject was excluded from further analyses. Download Figure 3-4, TIF file.

10.1523/ENEURO.0078-25.2025.f3-5 Figure 3-5 **VEP results: lateralized N1 latency.** Scatterplot showing the lateralized (C-I) N1 peak latencies in the foveola (x-axis) and perifovea (y-axis). Each dot represents a single subject. The dots scatter below the unity line (dashed line), indicating that N1 latencies in the perifovea were about 13  ms shorter than in the foveola (p < 0.001). Download Figure 3-5, TIF file.

10.1523/ENEURO.0078-25.2025.f3-6 Figure 3-6 **Global mean field power latency.** Left panel: grand average normalized GMFP for the foveola stimulation condition (blue) and perifovea stimulation condition (orange). Right panel: average (±1 sem) of the temporal lag between the two curves. A positive lag indicates an earlier perifoveal response, The inset shows the cross-correlation function averaged across all subjects (n = 18). The dashed curves indicate the point of zero correlation (horizontal line) and the zero-lag point (vertical line). The solid vertical line indicates the mean lag, around 10  ms, which confirms previous analyses and shows a shorter latency for perifoveal visual responses compared to foveal responses (p = 0.041). Download Figure 3-6, TIF file.

It has been suggested that the lateralization of the N1 peak is dependent on the vertical hemifield and on the eccentricity of stimulation (Kremláček et al., 2004). In particular, when the eccentricity of the stimulus is displayed at eccentricities higher than 17°, the N1 shows a paradoxical lateralization, with shorter latencies over the ipsilateral hemisphere (Kremláček et al., 2004). Even though our stimulation was at 6.5° of eccentricity, we controlled for possible ipsilateral effects. To this end, we performed the same peak analysis as in Fig. 3), on the ipsilateral N1 component (Extended Data Fig. 3-2). Results revealed that, similarly to what observed for the contralateral response, the ipsilateral N1 peak latency was about 10 ms shorter for the perifoveal than the foveal stimulation (two-tailed paired *t*-test: *t*(14) = 2.44, *p* = 0.028, BF_10_ = 2.38; Extended Data  Fig. 3-3).

Additionally, we performed a peak analysis on the lateralized N1, estimated over the difference between the contralateral and ipsilateral VEP responses. Contralateral N1 has been associated with stimulus amplification processes (Schindler et al., 2022) and was detectable in 17 out of 18 subjects (Extended Data Fig. 3-4). This analysis also confirmed a shorter latency for lateralized responses to perifoveal stimuli compared to foveal stimuli. This difference is approximately 13 ms (two-tailed paired *t*-test: *t*(16) = 5.23, *p* < 0.001, BF_10_ = 336.10; Extended Data Fig. 3-5), which is very similar to what was observed for the contralateral and ipsilateral N1 components.

Finally, we performed a peak-latency analysis based on the global mean field power (GMFP). The advantage of using GMFP for estimating the latency of an EEG response lies in its ability to capture the overall neural activity across the entire scalp, providing a robust and stable estimate of the latency across all cortical regions involved in the visual response. Specifically, we estimated the GMFP for stimuli presented to the right and left visual fields and normalized the values within the 0–200 ms interval. We then averaged the two responses to obtain the normalized GMFP shown in the panel on the left, which displays the grand-average normalized GMFP for the foveola stimulation condition (blue in Extended Data Fig. 3-6) and perifovea stimulation condition (orange in Extended Data Fig. 3-6). The latency of the two curves was estimated using the cross-correlation method. For each subject, we calculated the cross-correlation function between the GMFP at the two tested eccentricities and assessed the peak of the cross-correlation, which indicates the lag between the two curves. A positive lag indicates an earlier perifoveal response, while a negative lag indicates an earlier foveal response. This analysis confirmed that perifoveal visual responses were about 10 ms faster than the foveal ones (two-tailed paired *t*-test: *t*(17) = 2.21, *p* = 0.041, BF_10_ = 1.68; Extended Data Fig. 3-6).

Next, we explored whether larger discrepancies in N1 responses between the foveola and perifovea were associated with amplified differences in RTs. All analyses comparing RT and N1 used the RTs estimated from the same trials used to estimate N1. We found that the between-subject correlation between RTs and N1 was not statistically significant, neither when considering both eccentricities together (*F*(1, 28) = 0.23, *p* = 0.631, BF_10_ = 0.158, ${\text{R}}_{adj}^{2}=-0.02$, Pearson’s *ρ*(30) = 0.091; Fig. 4*A*), nor when examining them individually (foveola: *F*(1, 13) = 0.02, *p* = 0.878, BF_10_ = 0.196, ${\text{R}}_{adj}^{2}=-0.07$, Pearson’s *ρ*(15) = 0.043; perifovea: *F*(1, 13) = 0.045, *p* = 0.834, BF_10_ = 0.198, ${\text{R}}_{adj}^{2}=-0.07$, Pearson’s *ρ*(15) = 0.059). Overall, the unsigned correlation values were below 0.1. Similar results were obtained when the data were normalized to address individual variability (*F*(1, 13) = 0.03, *p* = 0.856, BF_10_ = 0.197, ${\text{R}}_{adj}^{2}=-0.07$, Pearson’s *ρ*(15) = 0.051; Fig. 4*B*). Our results suggest that there is no clear evidence of a strong correlation between N1 latency and reaction times, and the Bayes factor provided evidence for the absence of a correlation between RTs and N1 latencies (BF_10_ < 0.33). However, due to the small sample size (*N* = 15) our test is not sensitive enough to detect the presence of correlations smaller than 0.65 in data, with a power of 0.8. Further studies with much larger sample size (ideally >50) are necessary to determine if moderate or small correlations between these variables exists.

We additionally explored the within-subject correlation. This analysis revealed a significant but weak positive association between single-trial RT and N1 latency, with an average partial correlation coefficient of 0.08 ± 0.01 (mean ±1 SEM; *t*(12) = 4.31, *p* < 0.001, BF_10_ = 39.157). Notably, the within-subject partial correlation revealed a significant association in only 3 out of the 13 participants used in this analysis, confirming the weakness of the link between the two variables. As a control, we also measured the correlation between N1 and visual sensitivity, which proved to be non-significant (*F*(1, 13) = 2.55, *p* = 0.134, BF_10_ = 0.596, ${\text{R}}_{adj}^{2}=0.09$, Pearson’s *ρ*(15) = −0.405).

In summary, the findings reported here indicate a consistent influence of eccentricity on both RTs and N1 peak latencies. Specifically, we demonstrated an increase in both RTs and N1 latencies when stimuli are presented foveally compared to perifoveally. Nevertheless, the connection between these effects, whether examined in terms of inter-subject variability or intra-subject variations, remains unclear. This ambiguity suggests that the observed differences are likely mediated by distinct underlying processes.

## Discussion

The current study investigated the effects of stimulus eccentricity on both the timing of visual discrimination and the speed of visual-evoked responses. To this aim, we recorded manual choice RTs and EEG potentials evoked by visual stimuli presented either in the foveola (0.33°) or perifovea (6.5°). For the EEG responses, we focused on the latency of the N1 visual component, a reliable early visual component which is known to reflect the operation of discrimination processes (Vogel and Luck, 2000), to be modulated by spatial and object-based attention (Martinez et al., 2007), and to predict RT variability (Wastell and Kleinman, 1980; Antonova et al., 2016; Ribeiro et al., 2016).

Our study shows that both choice RTs and visual response latencies (as measured with the EEG as N1 latency) shorten when the stimulus is presented in the perifoveal region compared to when it is presented in the foveola. In other words, both manual reaction times and the dynamics of neural response appear to be faster as stimuli are presented away from the center of the visual field. At the eccentricities tested in the current study (i.e., 0.33° vs 6.5°), this speed-up is similar for RTs and N1 latencies, quantified at approximately 10–15 ms. However, a closer look at the relationship between these two effects reveals that they are largely independent of each other: both in terms of within and between subjects, the connection of the shifts in RT and in N1 latency is generally weak. This suggests that, despite their similarity, these two measures likely reflect the operation of relatively independent processes. However, both are influenced by a similar delay.

Overall, these results are in line with some behavioral (Carrasco et al., 2003, 2006; Poletti et al., 2017) and neurophysiological (Baseler and Sutter, 1997; Kremláček et al., 2004; Hagler, 2014) studies, which have demonstrated faster visual processing for more eccentric stimuli. However, to date, in both behavioral (Staugaard et al., 2016) and neurophysiological (Busch et al., 2004; Laron et al., 2009; Capilla et al., 2016) literature, the evidence is mixed, challenging the interpretation of such difference, as well as the identification of the neural mechanisms underlying it. A possible explanation for these mixed results may lie in fundamental differences in the visual stimulation and experimental procedures adopted across studies. For instance, Capilla et al. (2016) found that N1 latency evoked by a contrast-reversing check pattern, with size corrected for cortical magnification, was modulated by eccentricity. Specifically, the contralateral N1 in the lower visual hemifield peaked approximately 10 ms earlier in the fovea (0° to 2.6°) compared to the perifovea (2.6° to 9.8°). This difference in findings could be attributed to the distinct nature of the stimulation and the task. In Capilla’s study, multiple stimuli at varying eccentricities were presented simultaneously, and participants were not required to perform a rapid response task on the stimulus, as in the current study. Instead, they performed a secondary foveal task, which likely required the allocation of attention to the fovea. This attentional allocation could also contribute to the observed differences in latency between the two studies. In contrast, Busch et al. (2004) employed a paradigm more comparable to the one adopted in the current study, presenting black circles and squares on a white background for 250 ms and asking participants to identify the shape of the object. The stimuli were shown either in the fovea or at eccentricities of 4.3° and 8.6°, with a fixed size of 4°. They reported no effect of eccentricity on either RTs or N1 latency. It is plausible that the lack of eccentricity effects in their results stems from the fixed stimulus size, which would stimulate different numbers of neurons across eccentricities without being corrected for cortical magnification. Supporting this interpretation, other studies, such as Osaka and Yamamoto (1978), have demonstrated that when stimulus size is kept constant—thereby creating an imbalance in the number of neurons stimulated across tested eccentricities—both RTs and the latencies of visually evoked responses tend to increase with increasing eccentricity.

There are numerous factors that can, in theory, contribute to the emergence of a difference in processing times between foveal and peripheral stimuli. For example, it is known that the ratio between magno- and parvocells varies with the eccentricity (Connolly and Van Essen, 1984; Schein and de Monasterio, 1987; Azzopardi et al., 1999): the number of magnocells increases with increasing eccentricity, while the number of parvocells decreases. This different spatial distribution can lead to a different characterization of neural responses at different eccentricities. In particular, regarding the temporal characteristics of visual responses, it is well-established that the magnocellular pathway is characterized by faster neural responses compared to the parvocellular pathway, with a difference estimated to be between 15 and 20 ms, observable both in responses within the corresponding layers of the lateral geniculate body and in the primary visual areas of macaques (Nowak and Bullier, 1997; Schmolesky et al., 1998). Notably, this difference in neural latencies appears to be in the same range as the effect reported in the present experiment.

Beyond the magno/parvocell temporal characteristics, other structural differences could possibly explain the observed eccentricity effect. Recent evidence demonstrated that—even within the same neuron type (e.g., parvocells)—the neural response dynamic differs across eccentricities, with faster response observed in more peripheral neurons (Sinha et al., 2017; Bucci et al., 2025). This eccentricity effect may arise from intrinsic differences in the temporal response kinetics of photoreceptors (Sinha et al., 2017), axonal diameter (Bucci et al., 2025), myelination (Sanchez et al., 1986; FitzGibbon and Taylor, 2012), and/or circuitry differences across the visual field (Lamme and Roelfsema, 2000; Panzeri et al., 2001). For instance, in the fovea an abundance of recurrent (vs feedforward) circuits compared to the periphery, has been reported by Williams et al. (2008). Therefore, eccentricity-dependent temporal response difference might also reflect these circuitry differences.

Notably, since all these aforementioned mechanisms are low level and affect the earliest stages of visual processing, they likely influence both RTs and N1 latencies to a similar degree. In addition to these, there are also higher-level factors (e.g., decisional) which might differently affect RTs and N1 latencies. For instance, the relationship between RTs and spatial sensitivity—for the same retinal eccentricity—is greatly affected by the manipulation of decisional processes (Greenlee and Breitmeyer, 1989): while simple RTs increase with increasing spatial frequencies, choice RTs requiring more complex judgments (as the ones recorded in the present study) show a non-monotonic inverted u-shape function with longer response time at the point of maximal sensitivity of a typical contrast sensitivity function, at around 4 *c*/° (Greenlee and Breitmeyer, 1989). Interestingly, the stimuli used in the present study had an aperture of 0.06° and 0.25° (Fig. 1), thus the most informative spatial frequencies to discriminate the stimulus were approximately at 16 and 4 *c*/° in the foveola and perifovea, respectively. Based on Greenlee and Breitmeyer (1989)’s study, we should expect longer RTs for perifovea stimuli than foveal ones. However, we observe the opposite pattern of results. Therefore, the influence of decisional processes on the pattern of results observed in this study may not directly explain the major differences. Alternatively, this factor might reduce the size of the observed effect. This is in line with the reported larger effect size of 50–90 ms by Carrasco et al. (2003), where similar spatial frequencies were used at the tested foveal and peripheral eccentricities, and with results from Poletti et al. (2017), where simple RT were measured.

Here, we decided to focus on choice RT, as the previous literature suggests that the component of interest, namely the N1, is more pronounced in discrimination tasks (Vogel and Luck, 2000). Interestingly, despite similarities in motor preparatory processes (Miller and Low, 2001), both psychophysical (Greenlee and Breitmeyer, 1989) and electrophysiological studies (Dzianok et al., 2021) indicate that simple and choice RT tasks engage partially distinct neural mechanisms. Although the literature suggests that the eccentricity effect is also present in simple RT tasks (Poletti et al., 2017), it remains an open question whether the N1 latency effect observed here is also present when visual detection, rather than visual discrimination is engaged. More research is needed to address this issue.

Both RT and N1 are greatly affected by attention, and in particular by spatial attention mechanisms (Posner, 1980; Mangun, 1995). Here, we manipulated the deployment of spatial attention to investigate its effect on the eccentricity-related RT difference. Notably, allocating or not in advance spatial attention (i.e., blocked vs interleaved presentation of foveal and perifoveal trials, respectively) did not affect the overall eccentricity-dependent speed-up of RTs, suggesting that the effect is not influenced by attentional factors: even when attention is pre-allocated at the upcoming stimulus location, the RT effect does not change. This is in line with the evidence showing that covert attention does not eliminate speed of processing differences across eccentricity (Carrasco et al., 2006).

What could be the advantage in processing peripheral stimuli faster than foveal ones (or, conversely, processing foveal stimuli slower than peripheral ones)? In other words: what is the advantage of multi-speed vision? Peripheral vision often serves as an early warning system, detecting movement or changes in the surroundings before they reach the foveal area. By prioritizing the processing of peripheral stimuli, individuals can respond rapidly to potential dangers or opportunities, without needing to shift their gaze away from the central focus. While peripheral vision remains vigilant for any sudden changes, the foveal vision can dedicate more time and resources to scrutinizing critical information for decision making or navigation. This eccentricity effect likely reflects a generalized speed up of visual processing, which extends beyond foveal vs extrafoveal vision. In fact, previous studies examined different eccentricities (i.e., 0° vs 3° and 4° vs 9°), reporting similar effects as the one provided here.

In conclusion, our findings suggest that visual discrimination speeds vary across eccentricities, and this effect is accompanied by faster processing and shorter latency of early visual responses in the perifovea compared to the foveola. Nevertheless, the connection between the discrimination speed (as measured by the RT) and the visual processing speed (as measured by the N1 peak latency) is, at best, indirect, hinting at the involvement of many factors contributing to these delays.

## Synthesis

Reviewing Editor: David Franklin, Technische Universitat Munchen Decisions are customarily a result of the Reviewing Editor and the peer reviewers coming together and discussing their recommendations until a consensus is reached. When revisions are invited, a fact-based synthesis statement explaining their decision and outlining what is needed to prepare a revision will be listed below. The following reviewer(s) agreed to reveal their identity: Nina Hanning. Note: If this manuscript was transferred from JNeurosci and a decision was made to accept the manuscript without peer review, a brief statement to this effect will instead be what is listed below.

Your manuscript has been re-reviewed by the original reviewers. They both highlight the important step that you have done in measuring the evoked potentials and reaction times to stimuli presented at different eccentricities in a controlled setting. While the number of participants may not be large, this work may form the basis of further extensive work that could investigate the mechanisms driving these effects. While both reviewers appreciate the revisions to the manuscript, we have some suggestions for further revisions to the manuscript. Please use these to revise your manuscript for revision.

Reviewer 1

The authors have made minor revisions to their manuscript, partly addressing my previous points. Below I describe a problem I noticed with the authors newly provided power analysis and I also have a follow-up suggestions on subject exclusion. Beyond those points, the paper seems acceptable even though I am still doubtful that this manuscript adds more than a couple of (well-measured) data points to literature.

1. The addition of Bayes Factors is appreciated and useful - in particular with respect to the correlations reported. However, the power analysis the authors provided in their reply to my previous comment is not informative in its presented form. The authors base their estimation of the number of participants required (N = 783) on the correlation coefficient they obtained (rho = 0.1). But given the small sample they have (N = 15), this obtained coefficient is highly unreliable. I ran a simple simulation that revealed that with N = 15 and a true correlation of rho = 0.1, the resulting 95% confidence interval of that coefficient ranges from -0.44 to 0.59. In turn, even a true correlation of >0.5 (which would be considerable and of practical relevance), would clearly include rho = 0.1 in its 95% confidence interval.

I took the liberty and did the power calculation for the authors. The power of the currently reported correlation is indeed 0.065. Thus, the probability of a type II error (probability of not rejecting the false null hypothesis; H_0_ = 1-0.065) is 93.5%! This result strongly suggests that the correlation coefficient should not be trusted at all. In fact, to obtain a power of 1-H_0_ = 0.8 (as assumed in their calculations), the true correlation would have to be rho > 0.65 for N =15. The authors may want to check these results (e.g., here: https://webpower.psychstat.org/models/cor01/).

Based on these points, I suggest adding this information when arguing for the absence of a correlation. First, the authors should report the very low power of their tests (<0.065) and the correspondingly very high probability of a type II error (93.5%). Second, they should spell out that, with N = 15, the magnitude of the correlation that would be required to obtain a reasonable power (0.8). Third, their conclusion "Nevertheless, the practical relevance of such a weak correlation would remain questionable and would contribute little to the overall interpretation of the results." is certainly not warranted and should be deleted. In contrast, I think that their overall conclusion at the end of the Discussion hits the right tone: "the connection between discrimination speed (as measured by RT) and visual processing speed (as measured by N1 peak latency) is, at best, indirect, hinting at the involvement of many factors contributing to these delays."

2. I am still wondering if the study was pre-registered, but given that the authors have not replied to this question (despite it being asked twice), I assume it was not? They now report an a-priori defined threshold of 1 μV for exclusion of participants, which seems reasonable. For the sake of transparency, however, the authors should include individual VEP results for all participants (including those that were excluded) as an Extended Data figure for Figure 3.

Reviewer 2

The authors have thoroughly addressed all of my comments and made the necessary revisions. I find the pilot data presented in the rebuttal-submitted in response to my previous "significant" rating- compelling and would recommend including it in the manuscript, though I leave this decision to the authors.

## Author Response

Dear Dr. Benedetto:

I am pleased to inform you that your manuscript, "The speed of visual discrimination differs between foveola and perifovea: a combined EEG and behavioral investigation," has been judged potentially suitable for publication in eNeuro, provided appropriate revisions are made. The decision was a result of the reviewers and me coming together and discussing our recommendations until a consensus was reached. I have put together a fact-based synthesis statement explaining the decision and appended it to this email.

It is important that you revise your manuscript to address the reviewers' concerns and submit a point-by-point reply to each concern. Before making a final decision, I will carefully review your revisions and responses to the reviewers.

Yours sincerely, David Franklin Reviewing Editor eNeuro --------------------------------------------- Synthesis of Reviews:

Synthesis Statement for Author (Required):

Your manuscript has been re-reviewed by the original reviewers. They both highlight the important step that you have done in measuring the evoked potentials and reaction times to stimuli presented at different eccentricities in a controlled setting. While the number of participants may not be large, this work may form the basis of further extensive work that could investigate the mechanisms driving these effects. While both reviewers appreciate the revisions to the manuscript, we have some suggestions for further revisions to the manuscript. Please use these to revise your manuscript for revision.

We would like to sincerely thank the editor and the reviewers for their thoughtful evaluation of our manuscript. While we understand that the number of participants may not be large, we agree with the reviewers that this work is fundamental for further, more extensive studies that can investigate deeper into the mechanisms driving these effects. We are also grateful for their constructive feedback and suggestions for further revisions, which we have carefully addressed in the updated manuscript.

Below is a point-by-point reply to the reviewers' comments.

Reviewer 1 The authors have made minor revisions to their manuscript, partly addressing my previous points. Below I describe a problem I noticed with the authors newly provided power analysis and I also have a follow-up suggestions on subject exclusion. Beyond those points, the paper seems acceptable even though I am still doubtful that this manuscript adds more than a couple of (well-measured) data points to literature.

1. The addition of Bayes Factors is appreciated and useful - in particular with respect to the correlations reported. However, the power analysis the authors provided in their reply to my previous comment is not informative in its presented form. The authors base their estimation of the number of participants required (N = 783) on the correlation coefficient they obtained (rho = 0.1). But given the small sample they have (N = 15), this obtained coefficient is highly unreliable. I ran a simple simulation that revealed that with N = 15 and a true correlation of rho = 0.1, the resulting 95% confidence interval of that coefficient ranges from -0.44 to 0.59. In turn, even a true correlation of >0.5 (which would be considerable and of practical relevance), would clearly include rho = 0.1 in its 95% confidence interval.

I took the liberty and did the power calculation for the authors. The power of the currently reported correlation is indeed 0.065. Thus, the probability of a type II error (probability of not rejecting the false null hypothesis; H_0_ = 1-0.065) is 93.5%! This result strongly suggests that the correlation coefficient should not be trusted at all. In fact, to obtain a power of 1-H_0_ = 0.8 (as assumed in their calculations), the true correlation would have to be rho > 0.65 for N =15. The authors may want to check these results (e.g., here: https://webpower.psychstat.org/models/cor01/).

Based on these points, I suggest adding this information when arguing for the absence of a correlation. First, the authors should report the very low power of their tests (<0.065) and the correspondingly very high probability of a type II error (93.5%). Second, they should spell out that, with N = 15, the magnitude of the correlation that would be required to obtain a reasonable power (0.8). Third, their conclusion "Nevertheless, the practical relevance of such a weak correlation would remain questionable and would contribute little to the overall interpretation of the results." is certainly not warranted and should be deleted. In contrast, I think that their overall conclusion at the end of the Discussion hits the right tone: "the connection between discrimination speed (as measured by RT) and visual processing speed (as measured by N1 peak latency) is, at best, indirect, hinting at the involvement of many factors contributing to these delays." We thank the reviewer for the constructive feedback. We understand the concern that the estimated sample size based on a correlation coefficient of rho = 0.1 may not be informative given the small sample size of N = 15. Based on this, we revised our manuscript to include the following sentence:

1. ll 263-264: we changed the sentence "This lack of correlation persisted even..." with "Similar results were obtained when ..." 2. ll 265-271. We deleted the sentence: "Nevertheless, the practical relevance of such a weak correlation would remain questionable and would contribute little to the overall interpretation of the results."; and we included the following: "Our results suggest that there is no clear evidence of a strong correlation between N1 latency and reaction times, and the Bayes Factor provided evidence for the absence of a correlation between RTs and N1 latencies (BF10<0.33). However, due to the small sample size (N=15) our test is not sensitive enough to detect the presence of correlations smaller than 0.65 in data with a power of 0.8. Further studies with much larger sample size (ideally >50) are necessary to determine if moderate or small correlations between these variables exists." 2. I am still wondering if the study was pre-registered, but given that the authors have not replied to this question (despite it being asked twice), I assume it was not? They now report an a-priori defined threshold of 1 μV for exclusion of participants, which seems reasonable. For the sake of transparency, however, the authors should include individual VEP results for all participants (including those that were excluded) as an Extended Data figure for Figure 3.

To clarify, our study was not pre-registered. We apologize for not addressing this earlier in our previous responses.

Following your suggestion, we now include the individual VEP data for all participants as Extended Figure 3-1 in the revised manuscript (Extended Data). We also changed the text accordingly:

1. ll 187-190: "See also Extended Data (Figures 3-1:6) for additional analyses on the N1 peak latencies and individual curves, including ipsilateral N1 latencies, lateralized N1 latencies and global mean field power latencies (Kremlácek et al., 2004; Schindler, Busch, Bruchmann, Wolf, &Straube, 2022)" 2. Figure 3 caption: ". Individual curves and additional analyses are reported in the Extended Data, Figures 3-1:6." Extended Figure 3-1. Contralateral VEP responses in the foveola (blue) and perifovea (orange). Vertical bars indicate the contralateral N1 peak latency for the two eccentricity conditions (solid blue for the foveola and dashed orange for the perifovea). Each panel represents data from an individual subject. The contralateral N1 latency, for each subject and condition, was automatically determined using the MATLAB function findpeaks as the local negative peak occurring between 100 and 300 ms, with a peak prominence exceeding 1 μV. In three participants (represented by panels with dark curves), the N1 peak prominence fell below the predefined threshold in either the foveal or peripheral stimulation conditions, preventing a reliable assessment of the evoked response. Consequently, these subjects were excluded from further analyses.

Furthermore, we now include in Extended Data three additional analyses (Extended Figures 3-2:6) to corroborate the evidence of faster responses in the perifovea compared to the fovea. Notably, all three analyses confirmed the presence of faster responses for perifoveal vs foveal stimulation. (1) Ipsilateral N1 latencies. It has been suggested that lateralization of the N1 peak is dependent on the vertical hemifield and on the eccentricity of stimulation (Kremláček et al., 2004). In particular, when the eccentricity of the stimulus is displayed at eccentricities higher than 17{degree sign}, the N1 shows a paradoxical lateralization, with shorter latencies over the ipsilateral hemisphere (Kremláček et al., 2004). Even though our stimulation was at 6.5{degree sign} of eccentricity, we additionally controlled for possible ipsilateral effects. To this end, we performed the same peak analysis as in Figure 3 (Extended Figure 3-2), on the ipsilateral N1 component. Results revealed that, similarly to what observed for the contralateral response, the ipsilateral N1 peak latency was about 10 ms shorter for perifoveal than foveal stimulation (two-tailed paired t- test: t(14)=2.44, p=0.028, BF10=2.38, Extended Figure 3-3).

Extended Figure 3-2. Ipsilateral VEP responses in the foveola (blue) and perifovea (orange). Vertical bars indicate the ipsilateral N1 peak latency for the two eccentricity conditions (solid blue for the foveola and dashed orange for the perifovea). Each panel represents data from an individual subject. The ipsilateral N1 latency, for each subject and condition, was automatically determined using the MATLAB function findpeaks as the local negative peak occurring between 100 and 300 ms, with a peak prominence exceeding 1 μV. In three participants (represented by panels with dark curves), the N1 peak prominence fell below the predefined threshold in either the foveal or peripheral stimulation conditions, preventing a reliable assessment of the evoked response. Consequently, these subjects were excluded from further analyses.

Extended Figure 3-3. Scatterplot showing the ipsilateral N1 peak latencies in the foveola (x-axis) and perifovea (y-axis). Each dot represents a single subject.

The dots scatter below the unity line (dashed line), indicating that ipsilateral N1 latencies in the perifovea were about 10 ms shorter than in the foveola (p=0.028). (2) Lateralized N1 latencies. We additionally performed a peak analysis on the lateralized N1, estimated over the difference between the contralateral and ipsilateral VEP responses (C-I VEP). Contralateral N1 has been associated with stimulus amplification processes (Schindler, S., Busch, N., Bruchmann, M., Wolf, M. I., &Straube, T, 2022) and was detectable in 17 out of 18 subjects (Extended Figure 3-4). This analysis also confirmed a shorter latency for lateralized responses to perifoveal stimuli compared to foveal stimuli. This difference is approximately 13 ms (two-tailed paired t-test: t(16)=5.23, p<0.001, BF10=336.10, Extended Figure 3-5), which is very similar to what was observed for the contralateral and ipsilateral N1 component.

Extended Figure 3-4. Lateralized VEP responses in the foveola (blue) and perifovea (orange). Vertical bars indicate the lateralized N1 peak latency for the two eccentricity conditions (solid blue for the foveola and dashed orange for the perifovea). Each panel represents data from an individual subject. The lateralized N1 latency, for each subject and condition, was automatically determined using the MATLAB function findpeaks as the local negative peak occurring between 100 and 300 ms, with a peak prominence exceeding 1 μV. In one participant (represented by panels with dark curves), the N1 peak prominence fell below the predefined threshold in either the foveal or peripheral stimulation conditions, preventing a reliable assessment of the evoked response. Consequently, this subject was excluded from further analyses.

Extended Figure 3-5. Scatterplot showing the lateralized (C-I) N1 peak latencies in the foveola (x-axis) and perifovea (y-axis). Each dot represents a single subject. The dots scatter below the unity line (dashed line), indicating that N1 latencies in the perifovea were about 13 ms shorter than in the foveola (p<0.001). (3) Global mean field power. We performed an analysis to corroborate the evidence of faster responses in the perifovea using an additional method, namely the global mean field power (GMFP). The advantage of using GMFP for estimating the latency of an EEG response lies in its ability to capture the overall neural activity across the entire scalp, providing a robust and stable estimate of the latency across all cortical regions involved in the visual response. Specifically, we estimated the GMFP for stimuli presented to the right and left visual fields and normalized the values within the 0-200 ms interval. We then averaged the two responses to obtain the normalized GMFP shown in the panel on the left, which displays the grand average normalized GMFP for the foveola stimulation condition (blue) and perifovea stimulation condition (orange). The latency of the two curves was estimated using the cross-correlation method. For each subject, we calculated the cross-correlation function between the GMFP at the two tested eccentricities and assessed the peak of the cross-correlation, which indicates the lag between the two curves. A positive lag indicates an earlier perifoveal response, while a negative lag indicates an earlier foveal response. This analysis confirmed that perifoveal visual responses were about 10 ms faster than the foveal ones (two-tailed paired t- test: t(17)=2.21, p=0.041, BF10=1.68, Extended Figure 3-6).

Extended Figure 3-6. Left panel: grand average normalized GMFP for the foveola stimulation condition (blue) and perifovea stimulation condition (orange). Right panel: average ({plus minus}1 sem) of the temporal lag between the two curves. A positive lag indicates an earlier perifoveal response, The inset shows the cross-correlation function averaged across all subjects (n=18). The dashed curves indicate the point of zero correlation (horizontal line) and the zero-lag point (vertical line). The solid vertical line indicates the mean lag, around 10 ms, which confirms previous analyses and shows a shorter latency for perifoveal visual responses compared to foveal responses (p=0.041).

Reviewer 2 The authors have thoroughly addressed all of my comments and made the necessary revisions. I find the pilot data presented in the rebuttal-submitted in response to my previous "significant" rating- compelling and would recommend including it in the manuscript, though I leave this decision to the authors.

We sincerely thank the reviewer for their positive feedback and for recognizing the revisions we have made.

Regarding the suggestion to include the pilot data in the manuscript, we would like to clarify that these data are part of a larger ongoing study that we are currently finalizing. We believe it would be more beneficial to publish this dataset alongside the other data we are collecting. As such, we prefer to hold off on including the pilot data in this manuscript and reserve it for future publication once we have a more complete dataset.
