# Supplementary figures and images for: The Speed of Visual Discrimination Differs between Foveola and Perifovea: A Combined EEG and Behavioral Investigation
Source: eNeuro. 2025 Aug 8;12(8):ENEURO.0078-25.2025. doi: 10.1523/ENEURO.0078-25.2025 (PMC12345403; doi:10.1523/ENEURO.0078-25.2025)

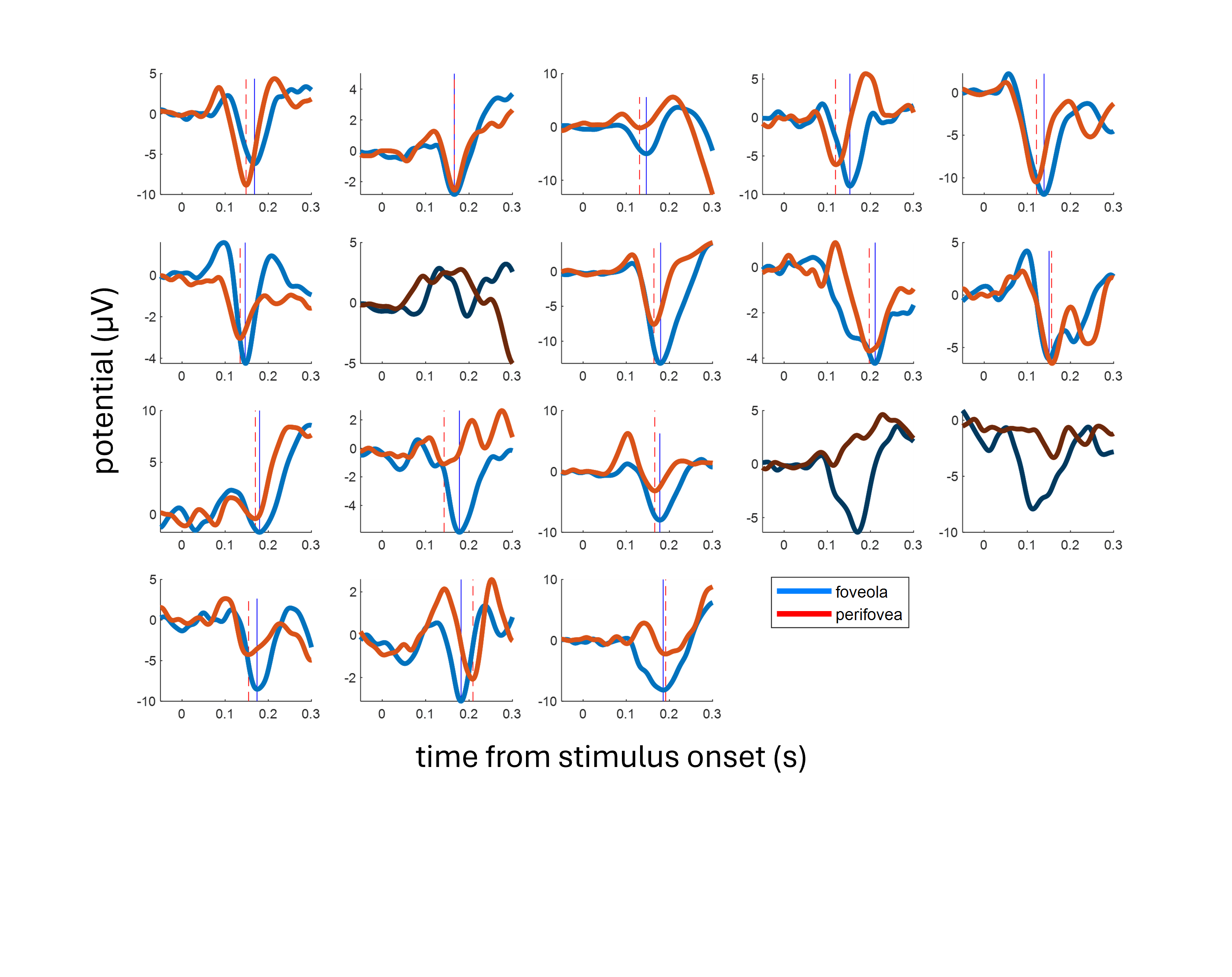

Supplement: Figure 3-1 — Contralateral VEP response. Contralateral VEP responses in the foveola (blue) and perifovea (orange). Vertical bars indicate the N1 peak latency for the two eccentricity conditions (solid blue for the foveola and dashed orange for the perifovea). Each panel represents data from an individual subject. The N1 latency, for each subject and condition, was automatically determined using the MATLAB function findpeaks as the local negative peak occurring between 100 and 300 ms, with a peak prominence exceeding 1 V. In three participants (represented by panels with dark curves), the N1 peak prominence fell below the predefined threshold in either the foveal or peripheral stimulation conditions, preventing a reliable assessment of the evoked response. Consequently, these subjects were excluded from further analyses. Download Figure 3-1, TIF file. [file eneuro-12-ENEURO.0078-25.2025-s003.tif]

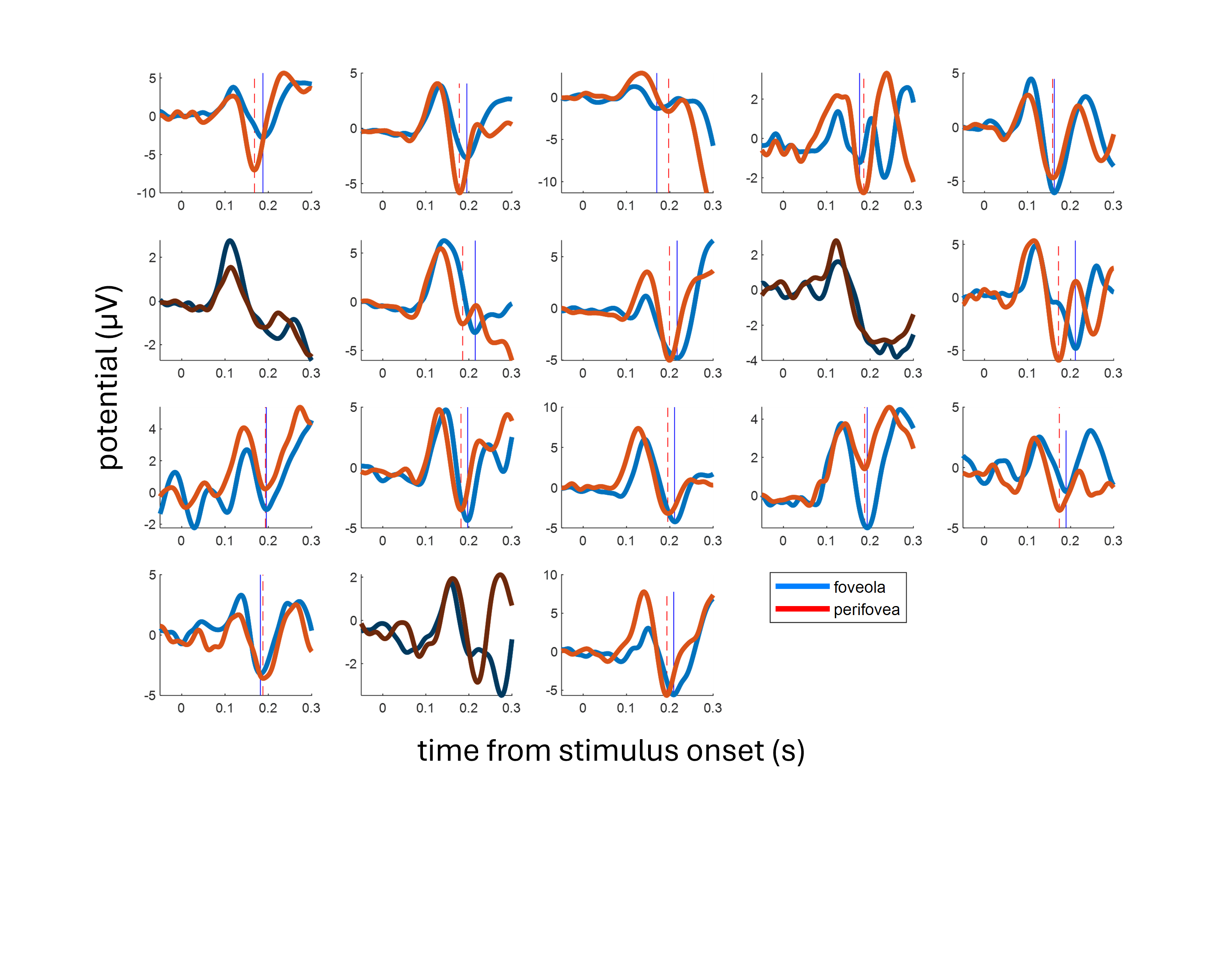

Supplement: Figure 3-2 — Ipsilateral VEP response. Ipsilateral VEP responses in the foveola (blue) and perifovea (orange). Vertical bars indicate the ipsilateral N1 peak latency for the two eccentricity conditions (solid blue for the foveola and dashed orange for the perifovea). Each panel represents data from an individual subject. The ipsilateral N1 latency, for each subject and condition, was automatically determined using the MATLAB function findpeaks as the local negative peak occurring between 100 and 300 ms, with a peak prominence exceeding 1 V. In three participants (represented by panels with dark curves), the N1 peak prominence fell below the predefined threshold in either the foveal or peripheral stimulation conditions, preventing a reliable assessment of the evoked response. Consequently, these subjects were excluded from further analyses. Download Figure 3-2, TIF file. [file eneuro-12-ENEURO.0078-25.2025-s004.tif]

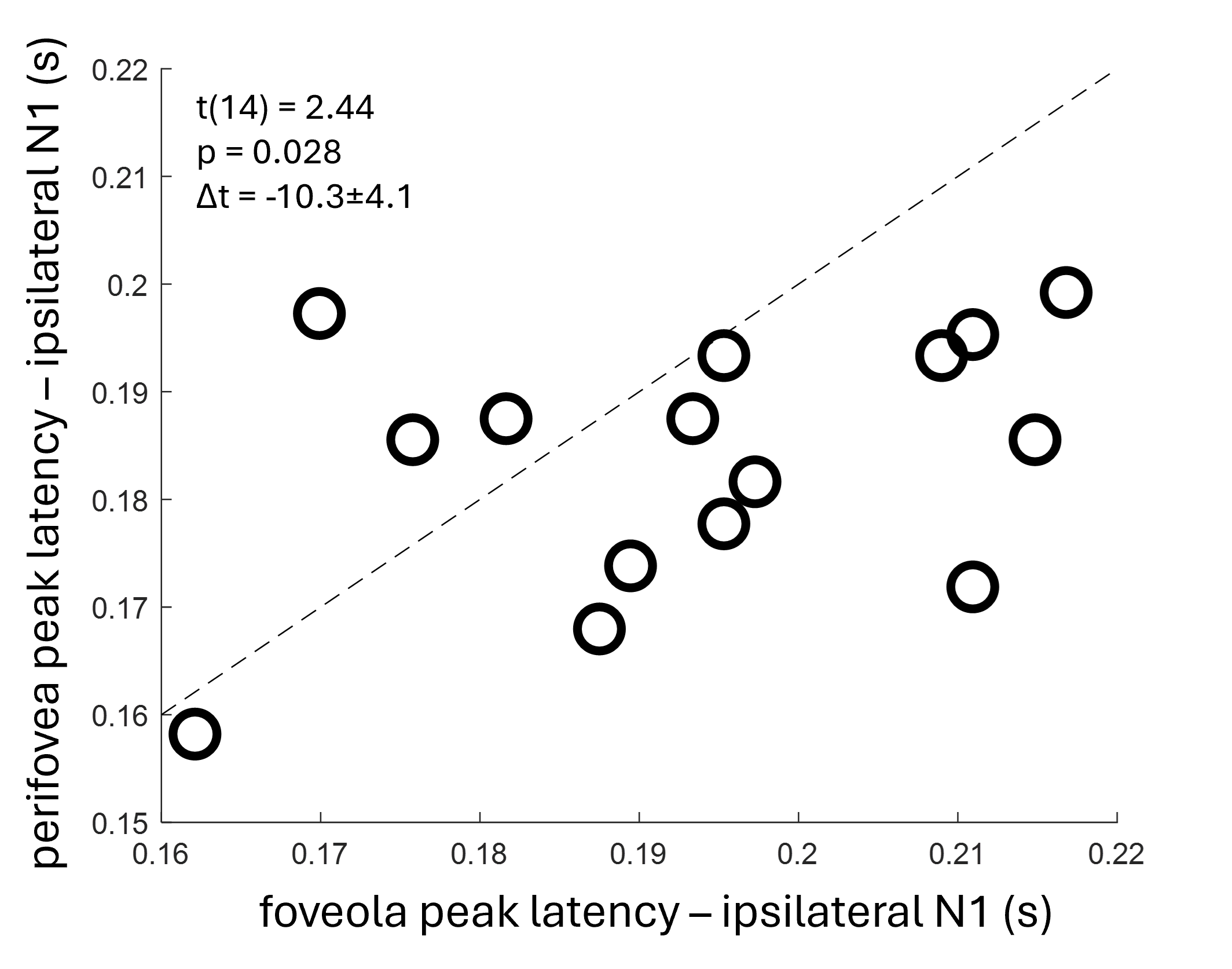

Supplement: Figure 3-3 — VEP results: ipsilateral N1 latency. Scatterplot showing the ipsilateral N1 peak latencies in the foveola (x-axis) and perifovea (y-axis). Each dot represents a single subject. The dots scatter below the unity line (dashed line), indicating that ipsilateral N1 latencies in the perifovea were about 10 ms shorter than in the foveola (p = 0.028). Download Figure 3-3, TIF file. [file eneuro-12-ENEURO.0078-25.2025-s005.tif]

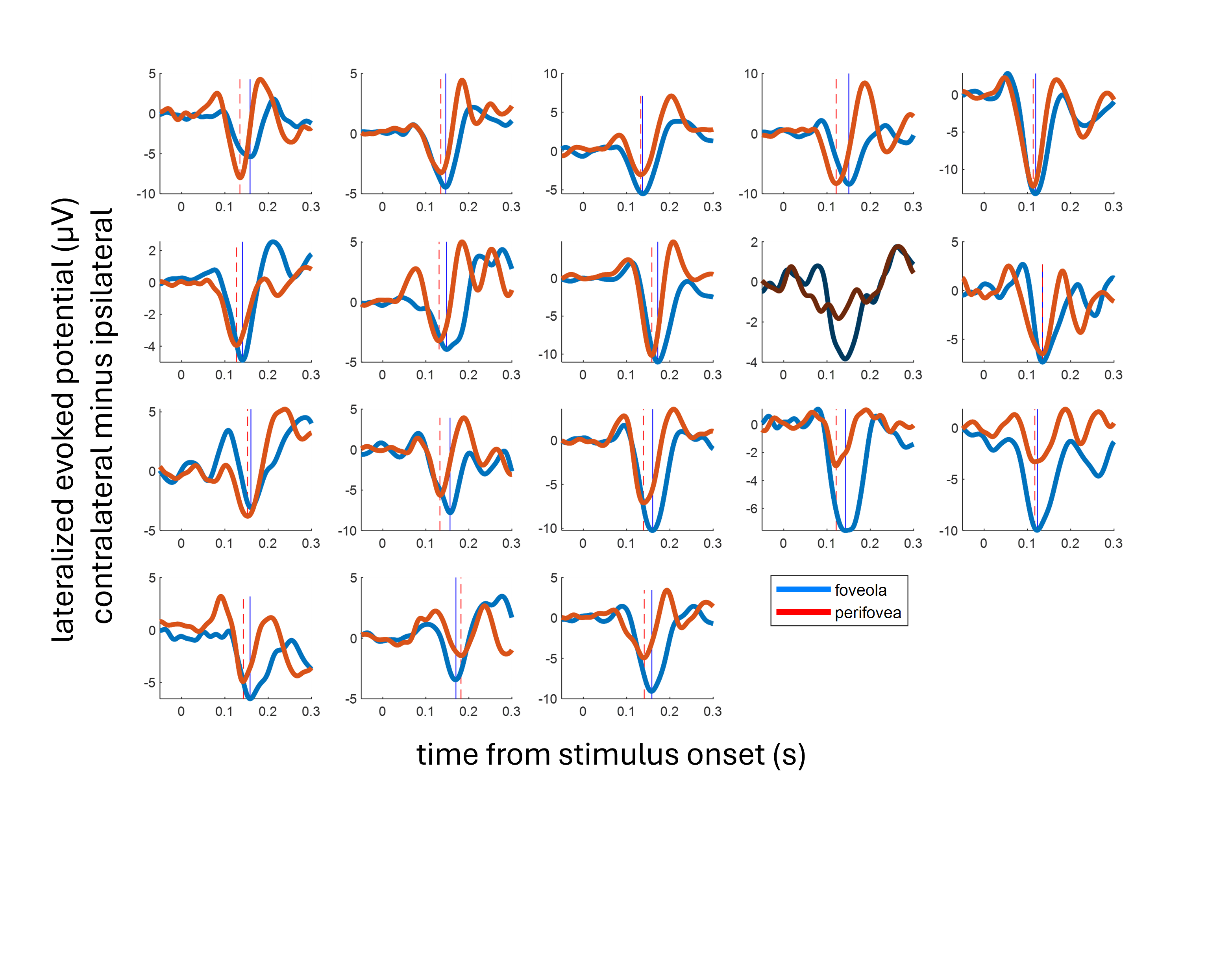

Supplement: Figure 3-4 — Lateralized VEP response. Lateralized VEP responses in the foveola (blue) and perifovea (orange). Vertical bars indicate the lateralized N1 peak latency for the two eccentricity conditions (solid blue for the foveola and dashed orange for the perifovea). Each panel represents data from an individual subject. The lateralized N1 latency, for each subject and condition, was automatically determined using the MATLAB function findpeaks as the local negative peak occurring between 100 and 300 ms, with a peak prominence exceeding 1 V. In one participant (represented by panels with dark curves), the N1 peak prominence fell below the predefined threshold in either the foveal or peripheral stimulation conditions, preventing a reliable assessment of the evoked response. Consequently, this subject was excluded from further analyses. Download Figure 3-4, TIF file. [file eneuro-12-ENEURO.0078-25.2025-s006.tif]

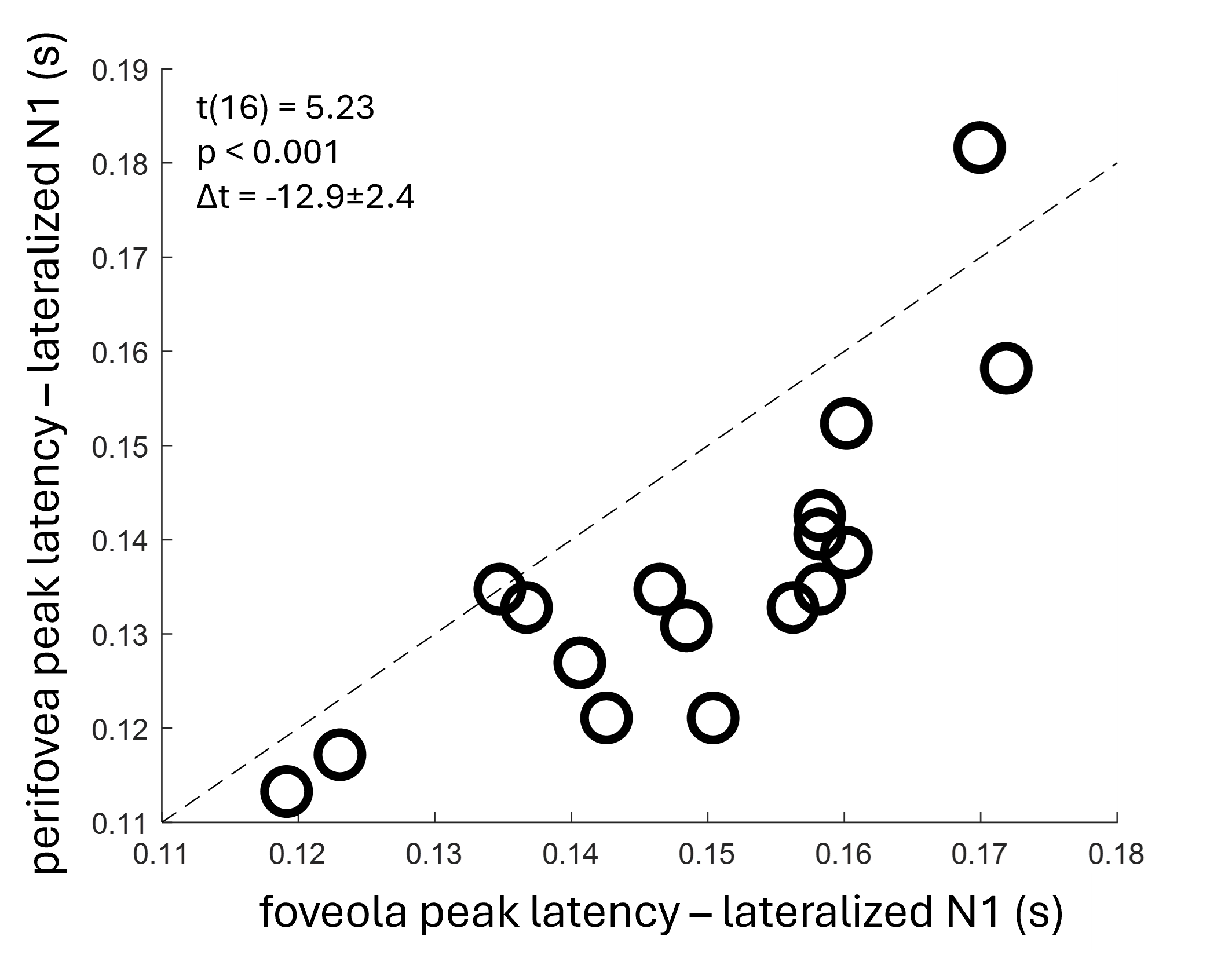

Supplement: Figure 3-5 — VEP results: lateralized N1 latency. Scatterplot showing the lateralized (C-I) N1 peak latencies in the foveola (x-axis) and perifovea (y-axis). Each dot represents a single subject. The dots scatter below the unity line (dashed line), indicating that N1 latencies in the perifovea were about 13 ms shorter than in the foveola (p < 0.001). Download Figure 3-5, TIF file. [file eneuro-12-ENEURO.0078-25.2025-s007.tif]

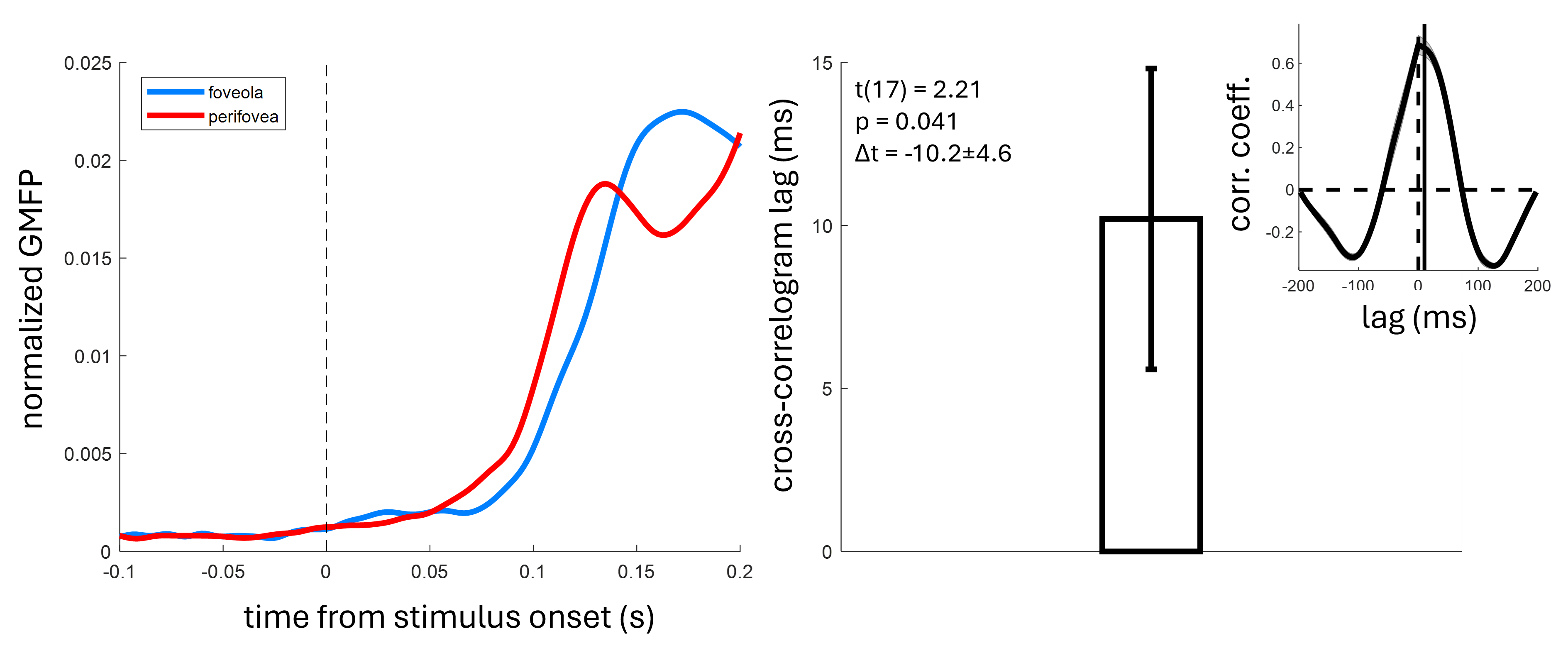

Supplement: Figure 3-6 — Global mean field power latency. Left panel: grand average normalized GMFP for the foveola stimulation condition (blue) and perifovea stimulation condition (orange). Right panel: average (±1 sem) of the temporal lag between the two curves. A positive lag indicates an earlier perifoveal response, The inset shows the cross-correlation function averaged across all subjects (n = 18). The dashed curves indicate the point of zero correlation (horizontal line) and the zero-lag point (vertical line). The solid vertical line indicates the mean lag, around 10 ms, which confirms previous analyses and shows a shorter latency for perifoveal visual responses compared to foveal responses (p = 0.041). Download Figure 3-6, TIF file. [file eneuro-12-ENEURO.0078-25.2025-s008.tif]
